# The use of low-calorie sweeteners is associated with self-reported prior intent to lose weight in a representative sample of US adults

**DOI:** 10.1038/nutd.2016.9

**Published:** 2016-03-07

**Authors:** A Drewnowski, C D Rehm

**Affiliations:** 1 Center for Public Health Nutrition, University of Washington, Seattle, WA, USA

## Abstract

**Background:**

Low-calorie sweeteners (LCSs) are said to be a risk factor for obesity and diabetes. Reverse causality may be an alternative explanation.

**Methods:**

Data on LCS use, from a single 24-h dietary recall, for a representative sample of 22 231 adults were obtained from 5 cycles of the National Health and Nutrition Examination Survey (1999–2008 NHANES). Retrospective data on intent to lose or maintain weight during the prior 12-months and 10-year weight history were obtained from the weight history questionnaire. Objectively measured heights and weights were obtained from the examination. Primary analyses evaluated the association between intent to lose/maintain weight and use of LCSs and specific LCS product types using survey-weighted generalized linear models. We further evaluated whether body mass index (BMI) may mediate the association between weight loss intent and use of LCSs. The association between 10-year weight history and current LCS use was evaluated using restricted cubic splines.

**Results:**

In cross-sectional analyses, LCS use was associated with a higher prevalence of obesity and diabetes. Adults who tried to lose weight during the previous 12 months were more likely to consume LCS beverages (prevalence ratio=1.64, 95% confidence interval (CI) 1.54–1.75), tabletop LCS (prevalence ratio=1.68, 95% CI 1.47–1.91) and LCS foods (prevalence ratio=1.93, 95% CI 1.60–2.33) as compared with those who did not. In mediation analyses, BMI only partially mediated the association between weight control history and the use of LCS beverages, tabletop LCS, but not LCS foods. Current LCS use was further associated with a history of prior weight change (for example, weight loss and gain).

**Conclusions:**

LCS use was associated with self-reported intent to lose weight during the previous 12 months. This association was only partially mediated by differences in BMI. Any inference of causality between attempts at weight control and LCS use is tempered by the cross-sectional nature of these data and retrospective self-reports of prior weight loss/maintenance intent.

## Introduction

People use low-calorie sweeteners (LCSs) to reduce dietary calories and manage body weight, with numerous randomized studies suggesting that their use can result in weight loss.^1, 2, 3, 4, 5, 6^ A position statement from the American Diabetes Association has included LCS use under nutrition recommendations and interventions for the management of diabetes.^4^ Systematic reviews of animal and human trials suggest that LCSs do not increase body weight or energy intake, and that in place of sugar, use of LCS leads to reduced energy intake and body weight.^7, 8^ Paradoxically then, some observational studies have reported that regular LCS use may lead to obesity, diabetes and the metabolic syndrome,^9, 10, 11, 12^ although the data were not always consistent.^13, 14, 15^ Missing from most studies evaluating the relation between LCS and deleterious outcomes was any information on past weight history or disease status, or the motivation to lose weight. When such information was available and accounted for, it tended to weaken such associations.^10^

People who are gaining weight may initiate LCS use for weight control, with varying degrees of success. People faced with the onset of type 2 diabetes may do the same, not only in an effort to lose/maintain weight, but also to avoid consumption of added sugars. The present analyses merged National Health and Nutrition Examination Survey (NHANES) dietary intake data with retrospective weight control histories, a rarely exploited resource within NHANES. The hypothesis was that current LCS use would be associated with self-reported weight loss/maintenance efforts and with 10-year weight fluctuations, including both weight gain and loss. A secondary hypothesis was that the association between past dieting (exposure) and current LCS use (outcome) would be observed for each LCS product type, including beverages, tabletop sweeteners and LCS foods. Third, we expected that the relation between self-reported prior dieting and LCS use would persist after adjusting for body mass index (BMI). The use of self-reported retrospective data on weight control history has many precedents. Numerous studies in the obesity and epidemiology literature have used self-reported weight histories to study the impact of past weight dynamics on current health outcomes.^16, 17, 18, 19^

## Materials and methods

### Population sample

The NHANES uses a complex, stratified, multistage probability sampling design; details of sample design and interview procedures have been published before.^20, 21^ NHANES provides data on dietary intakes and multiple health indicators for a nationally representative sample of children and adults in the United States.^20^ The analyses used data from five NHANES cycles: 1999–2000, 2001–2002, 2003–2004, 2005–2006 and 2007–2008. Included were data for 22 231 adults (⩾20 years) who were not pregnant, for whom height and weight data were available and who completed a valid 24-h dietary recall. The sample size was based on the availability of secondary data, but it was large enough to detect a very modest prevalence ratio (>1.076 or <0.925) between the exposure (weight loss intent (prevalence=36.2%)) and the outcome (use of LCS (prevalence=30.1%)) at α=0.05 with 95% power. All study protocols for NHANES 1999–2008 were approved by the institutional review board at the National Center for Health Statistics^22^ and informed consent was provided by all participants.

The NHANES 24-h recall uses the USDA (United States Department of Agriculture) Automated Multiple Pass Method, administered by trained interviewers. Respondents reported the types and amounts of all food and beverages consumed in the preceding 24-h, from midnight to midnight. Detailed methodology has been reported elsewhere.^20, 21^ Participant characteristics including age, gender, education and race/ethnicity were obtained from the demographic questionnaire. BMI (in kg m^−2^) was calculated using measured height and weight. Participants were divided into normal weight, overweight and obese, using standard BMI cut-points. Obese participants were divided further into class I obesity (BMI 30–34.9), class II obesity (BMI 35–39.9) and class III obesity (BMI ⩾40). The diagnosis of diabetes was determined via self-report and obtained from the NHANES diabetes questionnaire.

### Classification of LCS consumption by product category

The Food and Nutrient Database for Dietary Studies used to calculate energy and nutrient intakes in NHANES does not formally code foods and beverages containing LCSs.^23^ We therefore developed an algorithm to identify those foods and beverages that contained LCSs among ∼5700 items. Individual foods/beverages were queried based on their description, energy density (kcal per 100 g) and total/added sugars content. Three categories of LCS-containing foods/beverages were identified: beverages, foods and tabletop sweeteners, described further below. Categories of LCS-containing foods/beverages were created as we previously observed distinct age patterns in their use.^24^ The behavioral predictors of use may also vary across product category.

LCS beverages were defined as carbonated soft drinks, fruit drinks (not fruit juice), presweetened iced teas and sports and energy drinks labeled as being sugar free or low calorie. The most common LCS beverages were sugar-free cola, sugar-free fruit-flavored soft drink and fruit-flavored drink, made from low-calorie powder. The most frequently used tabletop LCSs were saccharin, sucralose and aspartame. Liquid LCSs were included in this category, but were infrequently consumed. Key LCS foods included yogurt, ice cream, grain-based desserts and candies. The level of detail in the food database did not permit for the evaluation of specific types of LCSs (for example, sucralose vs saccharin).

NHANES participants identified as LCS users were then assigned categories based on LCS consumption, namely: (1) consumers of LCS beverages (for example, diet soft drinks, diet fruit drinks, diet iced tea and low-calorie energy drinks), (2) consumers of LCS foods (for example, yogurt, ice cream, baked goods or candies), (3) consumers of tabletop LCS (for example, sucralose, aspartame, or saccharin) and (4) consumers of LCSs from at least one source, including multiple sources (beverages, foods or tabletop). An additional analysis categorized individuals by the number of LCS product types consumed (1 vs none or ⩾2 vs none) in an effort to measure intensity of LCS exposure.

### Weight history and weight control history

NHANES participants were asked if they tried to lose weight in the prior 12 months or if they tried to not grain weight (for example, maintain weight). An additional variable was created that summarized these two measures into a single measure of intent to lose/maintain weight. Beginning in 2005–-2006, adults who reported that they were trying to lose weight were not asked about trying to maintain their weight. For consistency, all data before 2005 were coded in a similar manner (that is, an individual could only be coded as trying to lose or maintain weight, but not both simultaneously). These questions provide a retrospective measure of weight loss/maintenance intent for the 12 months before the NHANES dietary survey.

Participants aged ⩾36 years were asked to self-report their weight at present and 10 years prior. These data were used to calculate 10-year weight change. Analyses of retrospective weight change was limited to adults <65 years of age to reduce the likelihood of including individuals experiencing age-related weight loss. Additional secondary analyses were conducted excluding individuals who experienced a health event that could result in unintended weight loss, including history of cancer (excluding non-melanoma skin cancer), liver disease, chronic bronchitis, emphysema or heart failure within 15 years of the interview.

### Statistical analysis

Descriptive analyses compared LCS users and nonusers in relation to obesity and diabetes status. Analyses were conducted for consumers of any LCS, by product category and for consumers of ⩾2 product types. As age, gender, race/ethnicity, socioeconomic status and smoking may confound any relation between LCS consumption and BMI and diabetes, a multivariable-adjusted analysis was conducted. For BMI, survey-weighted multinomial logistic regression models were fit with BMI category as the outcome and LCS category as the independent variable. For diabetes, a survey-weighted logistic regression model was fit. For both, a multivariable-adjusted model was fit that included age group, gender, race/ethnicity, family income and smoking status. In addition, the multivariable diabetes analysis adjusted for BMI category. Rather than present odds ratios, the marginal proportions were estimated representing the adjusted predicted prevalence of each outcome by LCS product type.

Additional analyses estimated the prevalence ratio of LCS use by weight control practices before and after adjusting for BMI category (<18.5, 18.5–24.9, 25–29.9, 30–34.9, 35–39.9 and ⩾40 kg m^−2^) using a survey-weighted generalized linear model of the Poisson family with a log-link adjusting for age group, gender and race/ethnicity.^25^ The extent by which BMI mediated the association between weight intent and LCS use was formally quantified by calculating the percent change in the log-prevalence ratio before and after including BMI in the model. A 95% confidence interval (CI) for the mediated effect was estimated using a bias-corrected bootstrap with 500 replications. Analyses of weight intent and LCS consumption was limited to individuals with data on BMI and weight loss (*n=*19 750) or loss/maintenance (*n=*21 049). For analyses with weight maintenance, individuals reporting intent to lose weight were excluded so that individuals trying to lose weight were not included in the unexposed group (*n=*14 528).

Lastly, the association between 10-year weight change and LCS use was evaluated. Because of anticipated nonlinear effects of weight change on the likelihood of LCS consumption, a restricted cubic spline with 3 knots was used.^26^ The analysis of 10-year weight change adjusted for age group, gender, race/ethnicity, education and self-reported height. Results are presented graphically and the prevalence ratio of LCS consumption compared with those who gained 10 lb over 10 years (the population median was +12 lb). To minimize the impact of outliers on the observed association, individuals with weight loss <1st percentile (−60 lb) or >99th percentile (+100 lb) were excluded from the graphs. All analyses were conducted in Stata 13 (College Station, TX, USA), accounting for the complex survey design of NHANES.

### Code availability

Stata code for the primary analysis is available from the authors.

## Results

Overall, LCSs of any type were consumed by 30.1% of adults, with more adults consuming LCS beverages (22.1%) than either tabletop LCS (11.4%) or LCS foods (4.6%). Approximately 23% of adults consumed only one type of LCS product, whereas 7.3% consumed two or more.

**Figure 1:**
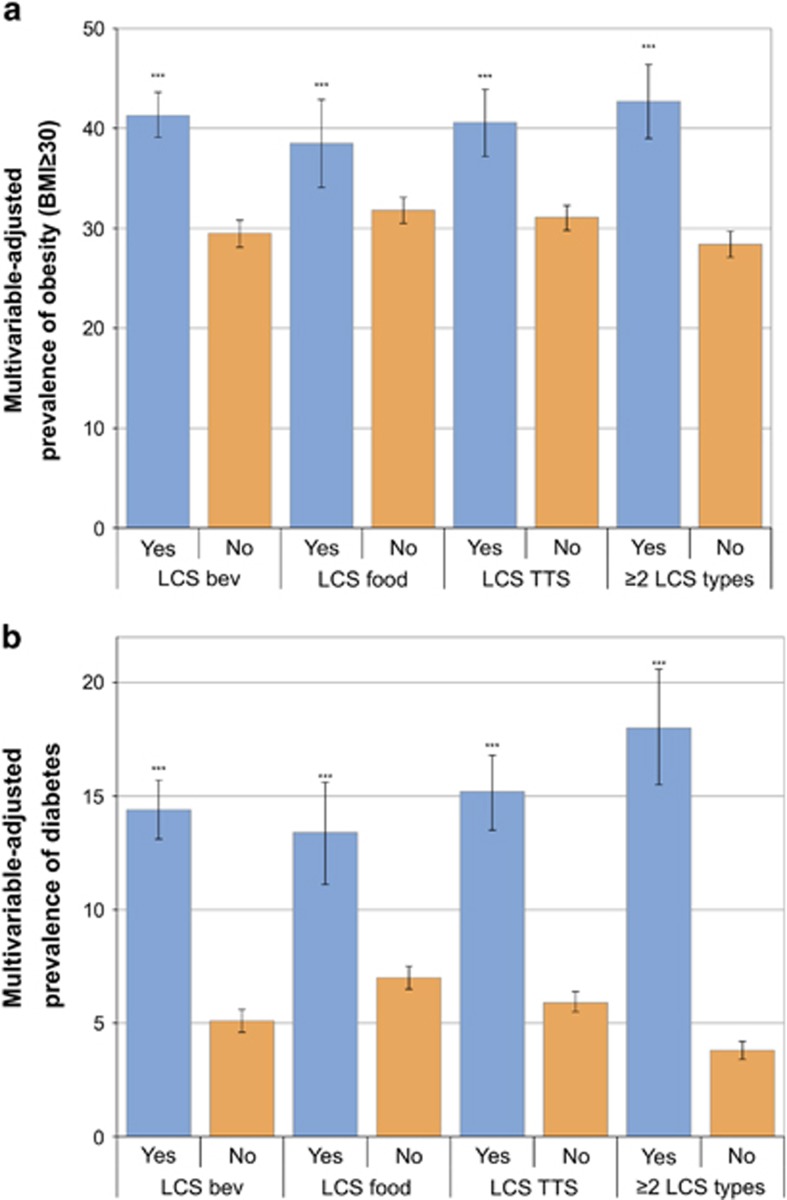
Multivariable-adjusted prevalence of obesity (**a**) and diabetes (**b**) by type of LCS consumed. *** indicates *P*-value<0.001 comparing LCS consumers to non-consumers.

In cross-sectional analyses, the expected relation between higher BMI and LCS use was observed, after adjusting for smoking and sociodemographic variables. The relation was significant for the entire population and separately for men and women (see Table 1). The relation between obesity (BMI ⩾30 kg m^−2^) and LCS consumption was significant for LCS beverages, tabletop LCS and LCS foods (see Figure 1a). Individuals consuming two or more types of LCSs were more likely to be obese than individuals consuming none (42.7% vs 28.4%) and were more likely to have class III obesity (7.3% vs 4.2%).

Table 2 shows the cross-sectional associations between LCS use and diabetes prevalence for the total population and separately for men and women. LCS use was significantly associated with higher diabetes prevalence (13.9% vs 4.0% for nonconsumers), after adjustment for age, gender, race/ethnicity, family income, smoking and BMI, and Figure 1b shows that the relation held for every product category.

Table 3 shows the relation between weight control history and LCS use, overall and by product type. Individuals who tried to lose weight during the past year were 64% (95% CI 54–75%) more likely to consume any type of LCS product after adjusting for age group, gender and race/ethnicity. The association between reported weight loss attempts and LCS use was observed for LCS beverages (72% more likely (95% CI 57–88%)), tabletop LCS (68% more likely (95% CI 46–91%)) and for LCS foods (93% more likely (95% CI 60–133%)). Individuals who tried to lose weight were 2.37 times as likely (95% CI 1.96–2.86) to consume two or more types of LCS products.

Similar results were obtained with the ‘trying to not gain weight' variable. The association between intent to lose or maintain weight and LCS use was stronger for men as compared with women, although overall women were more likely to report weight loss intent (44.2% vs 27.9%). Because diabetes is also strongly related to LCS consumption, sensitivity analyses examined the same relations after excluding individuals with diagnosed diabetes. In general, the relation between weight history and LCS use was stronger, rather than weaker, after excluding individuals with diabetes.

Adjustment for BMI had very little impact on the association between weight control history and LCS use, as shown in Table 3. Overall, the association between prior intent to lose or maintain weight and current LCS use was robust to adjustment for six levels of BMI and remained statistically significant. In separate analyses by gender and LCS product type, BMI appeared to be a partial mediator of the association. Specifically, for weight loss intent and total LCS consumption among men, BMI explained 32.4% (95% CI 22.4–42.4%) of the association. Similar effects were observed for LCS beverages and tabletop LCS. The mediating effect of BMI was much weaker in women as compared with men.

**Figure 2:**
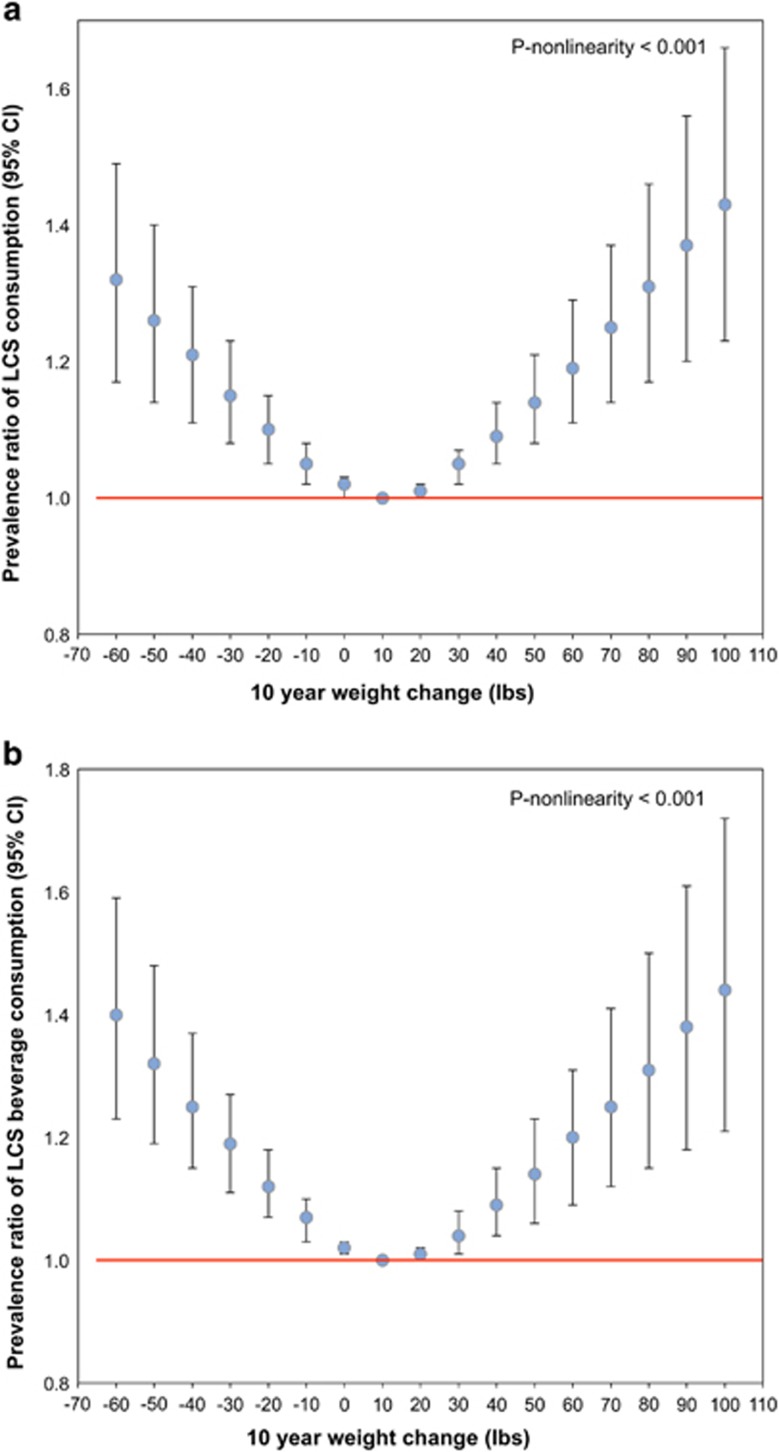
Multivariable-adjusted prevalence ratio between 10-year weight change and consumption of any LCS (**a**) and LCS beverages (**b**).

**Figure 3:**
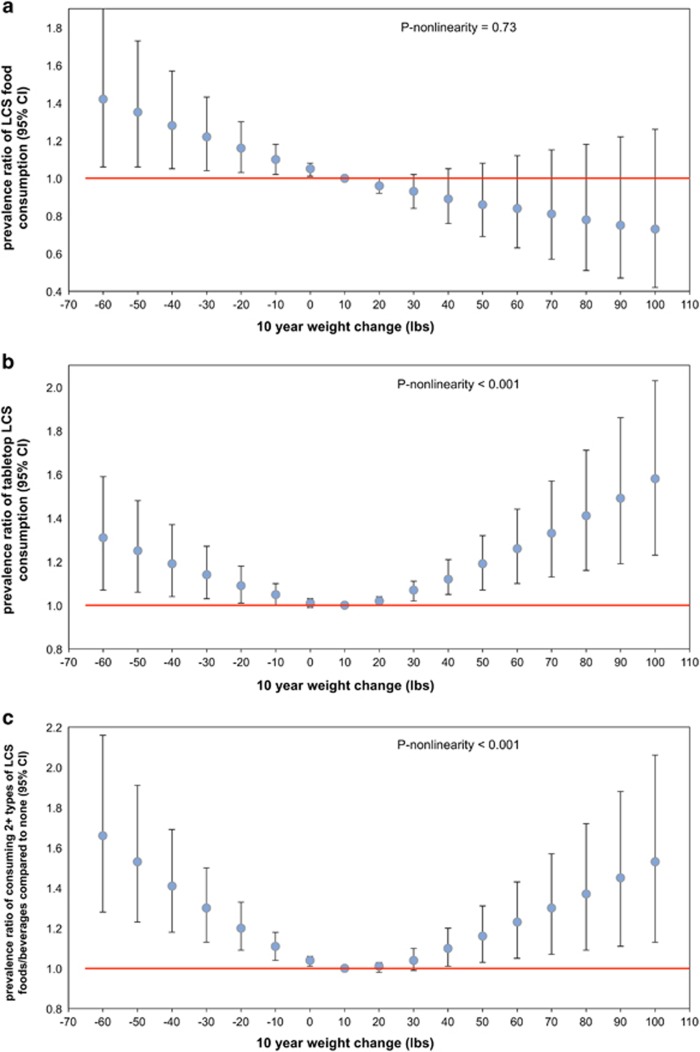
Multivariable-adjusted prevalence ratio between 10-year weight change and consumption of LCS foods (**a**), tabletop LCS (**b**) and ⩾2 LCS product types (**c**).

Current self-reported weight was highly correlated with measured weight (*r*=0.975). In addition, retrospectively reported weight at age 18 among young and middle-aged adults appeared to be valid, with correlation coefficients for recalled versus measured prior weight to be 0.87 for both men and women.^27, 28^ Figures 2 and 3 show the association between retrospective 10-year weight change and current LCS consumption. LCS use was much more common among individuals who experienced significant weight change in the preceding 10 years as compared with those who did not. A notable nonlinear relationship between prior significant weight change and current LCS consumption was observed for total LCS and all LCS types (*P*-nonlinearity<0.001), excluding LCS foods (*P*-nonlinearity=0.73). Compared with individuals who gained only 10 lb over the prior 10 years, individuals showing greater weight fluctuations (gaining and losing) were more likely to be current LCS consumers, adjusting for age group, gender, race/ethnicity, self-reported height and education.

The association between the 10-year weight change and consuming two or more types of LCS was particularly strong. Individuals who lost 50 lb in the prior 10 years were 47% (95% CI 19–83%) more likely to consume two or more LCS products, whereas those who gained 50 lb were 13% (95% CI −1 to 27%) more likely to consume two or more LCS products as compared with individuals who gained 10 lb over the prior 10 years. Individuals who lost 50 lb were 26% (95% CI 14–40%) more likely to use any LCS; those who gained 50 lb were 14% (95% CI 8–21%) more likely to consume LCS. Additional analyses excluding the 9.5% of adults aged 36–64 years who experienced cancer (excluding non-melanoma skin cancer), liver disease, chronic bronchitis, emphysema or congestive heart failure in the prior 15 years did not alter results (data not shown).

## Discussion

That obese individuals are more likely to use LCS beverages and foods than are normal-weight individuals is well established.^24^ However, the chronology of this association is not. Some suggest that LCS may have caused weight gain by promoting sweet taste preferences and causing people to eat more.^12, 29, 30^ Others suggest reverse causality, such that overweight and obese people turn to LCSs to manage body weight.^31^ Similar debates abound for the role of LCS products and diabetes. Some researchers have suggested that LCSs promote type 2 diabetes and the metabolic syndrome; others point to the use of LCS in diabetes management.^4, 12, 13^ A recent meta-analysis and systematic review of prospective studies observed LCS beverage consumption to be associated with a modest increased risk of diabetes, but could not rule out that publication bias and/or residual confounding explained the association, summarizing that the prospective evidence was of ‘low quality'.^32^ The dilemma is not readily resolved, given that many of the observations have been based on cross-sectional data or prospective observational studies with limited information on weight history.^9^

Merging a number of NHANES databases allowed us to gain some insight into the relation between dieting behaviors over the previous 12 months and current LCS use. The NHANES surveys do, in fact, include some retrospective data on weight management. Here, self-reported attempts at weight loss or maintenance were the measures of prior 12-month exposure, whereas LCS use, adjusted for BMI, was the principal outcome variable of interest. This study provides the first analysis of the association between current LCS use, past weight loss/maintenance intent and 10-year weight history. Using 24-h dietary recall from NHANES, we were able to classify LCS consumers by product category: LCS beverages, tabletop LCS and LCS foods. Many past analyses of LCS use did not or could not distinguish among product categories or were limited to LCS beverages only.

Trials and observational studies alike have tended to focus on low-calorie beverages.^5, 9, 10, 13, 15^ Should the behavioral predictors of use or the effects on weight management/health differ by LCS category, the focus on LCS beverages may conceal the global impact of LCSs.^33^ Notably, we observed some heterogeneity in the strength of the association between weight loss/maintenance intent and LCS product type, the association being strongest for LCS foods, particularly among men. The reasons for the stronger association between weight loss/maintenance intent and LCS foods are unclear. One potential explanation is that during this time period (1999–2008) LCS foods represented a unique, and also less common, alternative to other sweetened foods (for example, grain-based deserts, ice cream or candy) that were more likely to be chosen by individuals trying to control their weight. The lower prevalence of LCS food consumption as compared with tabletop and LCS beverage consumption provides some evidence that this may be the case. As the number of LCS products has increased, revisiting this association using current data may be informative.^34^

Those NHANES participants who reported trying to lose or not gain body weight during the previous 12 months were much more likely to use LCSs. These associations between prior attempts at weight control and current LCS use held for both men and women and were observed for LCS beverages, tabletop LCS and LCS foods. The associations were robust in mediation analyses, after adjusting for BMI. In other words, the relation between weight loss/maintenance intent and current LCS use was not unique to obese individuals but held at all levels of BMI. That would suggest that LCS use was tied directly to dieting behaviors, regardless of whether the participants were overweight or obese. This new finding suggests reverse causality, linking LCS use with trying to lose or maintain body weight. Furthermore, the association between weight loss/maintenance intent and LCS use was stronger among men than women. A likely explanation is that the prevalence of both LCS use and the likelihood of trying to lose/maintain was lower among men than women, and this would tend to cause relative measures of association to be stronger than when the exposure and outcome are more common.

The inclusion of NHANES retrospective data on past attempts to manage body weight provided the critical motivational component missing from cross-sectional evaluations of LCS use and weight. Even though retrospective cohort studies^35^ rank low on the evidence hierarchy, especially when based on self-report, such data can be valuable when obtained for a large and nationally representative population of US adults. Within those constraints, trying to lose weight was one likely predictor of current LCS use. Given that current LCS consumers were more likely to be obese, one possibility is that individuals consuming LCS beverages and foods may have switched to diet beverages after having gained weight.^1, 10^

Analysis of self-reported 10-year weight change provided additional support for this hypothesis. Greater weight fluctuations were associated with LCS use. It is well known that prior weight loss is one of the strongest predictors of subsequent weight gain.^36^ It is therefore possible that those who lost weight in the prior 10 years turned to LCSs to reduce the probability of subsequent weight gain. On the other hand, individuals who experienced weight gain might turn to LCSs in an effort to lose weight or halt weight gain. Interestingly, the nonlinear association observed for overall LCS consumption, LCS beverages and tabletop LCS was not observed for LCS foods. Individuals who lost weight in the prior 10 years were more likely to consume LCS foods, whereas individuals gaining weight were less likely. Along with the stronger results for the association between weight loss/maintenance and LCS food consumption, there may be some unique aspects of LCS food consumption that merit further attention.

The finding that past weight fluctuations are a predictor of LCS use supports the hypothesis that people troubled by weight gain may turn to LCS as a strategy for weight control.^31^ Authors of prospective observational studies have noted that the most likely explanation for their observation of an association between LCS beverage and long-term weight gain was that LCS beverage use may be a marker for individuals already on a weight gain trajectory.^9^ An opposing view has been that it is LCS use that precedes any weight gain through a variety of metabolic and physiological mechanisms.^14, 37, 38^ Alternative mechanisms invoked the weakening of learned responses secondary to LCS use and the inability to accurately estimate energy needs.^13^

In previous analyses of NHANES data, LCS use was strongly related to age, gender, race/ethnicity and socioeconomic status. LCS consumers were more likely to be female, non-Hispanic white, better educated and to have higher household incomes.^24^ LCS consumers also tended to have healthier diets and be more physically active.^24, 39^ Such data run counter to the hypothesis that LCS use directly leads to obesity and diabetes, as the sociodemographic groups that consume the most LCSs tend to be those with the lowest prevalence of obesity and diabetes. On the other hand, age is associated with higher LCS use and with higher rates of obesity and diabetes.

The study had several strengths. First, the NHANES data provided us with a large and nationally representative sample of US adults making results comparable to other recent studies. Second, BMI was calculated using measured heights and weights, reducing concern regarding measurement error in the analysis treating BMI as a mediator of the weight intent and LCS consumption associations. Third, this was one of few studies to assign LCS consumers to different product categories. Most studies have focused on LCS beverages, the leading product category. This classification scheme may improve our understanding of the contextual, behavioral and environmental influences on LCS consumption. Finally, the joining of dietary and weight history variables allowed us to address some potential motivations behind LCS use.

Several limitations also need to be noted. First, NHANES dietary intake and health data are cross-sectional and no assignments of causality can be made with regard to dietary intakes and health outcomes. Second, the dietary intake data were based on a single 24-h dietary recall that does not fully reflect habitual intakes and underestimates the proportion of individuals consuming a given food or beverage.^40^ Third, although we were interested in comparing various types of LCS products, people using one type of LCS product are more likely to use other types (for example, among LCS beverage consumers, 23.5% consumed tabletop LCS compared to 8.0% of nonconsumers), making it challenging to independently evaluate a single product type. Third, weight control history was based on retrospective self-report and is potentially subject to random and systematic measurement error. The accuracy of self-reported weight loss/maintenance intent may be influenced by age, gender or body weight. The temporal relation between the weight loss/maintenance intent and diet was also unclear. In other words, although participants reported attempts to lose/maintain weight over the previous 12-month period, there is no information on when those attempts began. In addition, the analysis of retrospectively assessed weight change for purposes of comparability relied on self-reported weights for both the current and historical measurement, though it has previously been shown to be valid and is used much in the literature.

In summary, few population-based studies on LCS use have included any data on past weight management practices or weight trajectories before baseline. As use of LCS products appears to be on the rise and is an oft-employed weight loss/management strategy, understanding the potential risks and benefits of their use is critical.^24, 41^ The present analyses suggest that trying to lose or maintain body weight over a 12-month period was associated with higher LCS use, independent of body weight. However, additional prospective studies that carefully measure past weight history and weight management efforts are needed to better understand the impact of LCS use on weight on free-living populations.

## Figures and Tables

**Table 1:** Multivariable-adjusted^a^ prevalence of underweight, healthy weight, overweight and obesity by LCS use versus nonuse for the total population and by gender, NHANES 1999–2008

	N	*Any LCS*	*No LCS*	P-*value*^b^
*Total population*				
Underweight (<18.5)	312	0.8 (0.1)	2.1 (0.2)	<0.001
Healthy weight (18.5–24.9)	5850	24.1 (0.8)	35.4 (0.6)	<0.001
Overweight (25–29.9)	7090	34.3 (1.1)	34.2 (0.5)	0.91
Obese (⩾30)	6763	40.8 (1.1)	28.4 (0.7)	<0.001
Class I obesity (30–34.9)	3952	21.4 (0.8)	17.6 (0.4)	<0.001
Class II obesity (35–39.9)	1707	11.4 (0.6)	6.6 (0.3)	<0.001
Class III obesity (⩾40)	1104	8.0 (0.5)	4.1 (0.2)	<0.001
				
*Women*				
Underweight (<18.5)	196	1.2 (0.2)	2.9 (0.3)	<0.001
Healthy weight (18.5–24.9)	2997	30.3 (1.0)	39.0 (1.0)	<0.001
Overweight (25–29.9)	2971	28.6 (1.2)	28.1 (0.8)	0.33
Obese (⩾30)	3733	39.9 (1.1)	30.0 (0.9)	<0.001
Class I obesity (30–34.9)	1961	18.9 (0.9)	16.6 (0.6)	<0.001
Class II obesity (35–39.9)	1029	12.1 (0.7)	7.9 (0.4)	<0.001
Class III obesity (⩾40)	743	9.0 (0.6)	5.5 (0.4)	<0.001
				
*Men*				
Underweight (<18.5)	116	0.2 (0.1)	1.3 (0.2)	0.012
Healthy weight (18.5–24.9)	2853	16.8 (1.1)	31.4 (0.7)	<0.001
Overweight (25–29.9)	4119	40.3 (1.5)	40.5 (0.6)	0.46
Obese (⩾30)	3030	42.7 (1.6)	26.7 (0.8)	<0.001
Class I obesity (30–34.9)	1991	24.3 (1.2)	18.9 (0.9)	<0.001
Class II obesity (35–39.9)	678	10.9 (0.8)	5.2 (0.4)	<0.001
Class III obesity (⩾40)	361	7.4 (0.8)	2.7 (0.3)	<0.001

Abbreviations: LCS, low-calorie sweetener; NHANES, National Health and Nutrition Examination Survey.

a Adjusted for age group, gender (total model), race/ethnicity, family income-to-poverty ratio and smoking status.

b Comparing prevalence of each weight category among consumers and nonconsumers.

**Table 2:** Age-adjusted and multivariable^a^ prevalence of diabetes by LCS use versus nonuse, NHANES 1999–2008

	*N*	*Any LCS*	*No LCS*	P-value *of difference*^a^
Age adjusted				
Total population	22 217	14.4 (0.6)	4.3 (0.2)	<0.001
Women	11 040	12.2 (0.7)	4.7 (0.3)	<0.001
Men	11 117	16.9 (0.8)	3.9 (0.3)	<0.001
				
Multivariable adjusted^a^				
Total population	20 005	13.9 (0.6)	4.0 (0.2)	<0.001
Women	9892	11.2 (0.7)	4.0 (0.3)	<0.001
Men	10 113	17.1 (0.8)	4.0 (0.3)	<0.001
				
Multivariable adjusted (age⩾50 years)^a^				
Total population	9852	24.4 (1.0)	7.5 (0.5)	<0.001
Women	4905	19.7 (1.3)	7.4 (0.6)	<0.001
Men	4947	30.0 (1.4)	7.5 (0.6)	<0.001

Abbreviations: LCS, low-calorie sweetener; NHANES, National Health and Nutrition Examination Survey.

a Adjusted for age group, gender (total model), race/ethnicity, family income-to-poverty ratio, smoking status and 6 categories of body mass index (BMI; <18.5, 18.5–24.9, 25–29.9, 30–34.9, 35–39.9 and ⩾40 kg m^−2^).

**Table 3:** Prevalence ratio of consuming LCS by weight intention, before and after adjusting for BMI, and percent of association between weight intention and LCS consumption explained by BMI

	*Overall*			*Men*			*Women*		
	*Model 1*^a^	*Model 2*^b^	*% Mediated by BMI (95% CI)*^c^	*Model 1*^b^	*Model 2*^b^	*% Mediated by BMI (95% CI)*^c^	*Model 1*^b^	*Model 2*^b^	*% Mediated by BMI (95% CI)*^c^
*Lose weight*									
Total LCS	1.64 (1.54, 1.75)	1.49 (1.39, 1.61)	18.6 (14.7, 24.1)	1.72 (1.55, 1.90)	1.48 (1.42, 1.66)	32.4 (24.4, 42.4)	1.56 (1.45, 1.68)	1.45 (1.34, 1.57)	16.4 (11.5, 24.2)
LCS beverage	1.72 (1.57, 1.88)	1.54 (1.4, 1.7)	20.3 (14.6, 26.7)	1.78 (1.55, 2.05)	1.50 (1.28, 1.74)	30.4 (21.1, 42.1)	1.64 (1.49, 1.80)	1.51 (1.37, 1.67)	16.5 (9.5, 25)
LCS food	1.93 (1.60, 2.33)	1.89 (1.55, 2.31)	1.5 (−7.7, 11.1)	2.54 (1.84, 3.51)	2.50 (1.72, 3.64)	1.4 (−16.9, 23.6)	1.67 (1.31, 2.13)	1.62 (1.26, 2.08)	5.0 (−9.1, 21.1)
LCS tabletop	1.68 (1.47, 1.91)	1.53 (1.33, 1.75)	22.1 (14.8, 29.9)	1.82 (1.54, 2.15)	1.54 (1.28, 1.86)	28.6 (16.6, 44.5)	1.55 (1.29, 1.85)	1.45 (1.21, 1.74)	25.3 (12.8, 44.8)
									
*Number of LCS*									
1 vs none	1.63 (1.5, 1.78)	1.47 (1.35, 1.61)	21.2 (15.4, 27.7)	1.66 (1.47, 1.88)	1.42 (1.23, 1.63)	31.2 (21.1, 45.6)	1.58 (1.44, 1.72)	1.46 (1.33, 1.59)	17.4 (9.9, 25.7)
⩾2 vs none	2.37 (1.96, 2.86)	2.09 (1.72, 2.54)	14.5 (9.2, 21)	2.85 (2.19, 3.7)	2.23 (1.69, 2.95)	23.3 (15, 35.7)	2.07 (1.62, 2.66)	1.89 (1.48, 2.42)	12.7 (5.3, 21.4)
									
*Lose or maintain weight*									
Total LCS	1.67 (1.56, 1.79)	1.54 (1.43, 1.65)	16.4 (12.6, 21.1)	1.73 (1.56, 1.91)	1.51 (1.35, 1.68)	24.8 (17.3, 33.2)	1.60 (1.48, 1.73)	1.50 (1.39, 1.62)	13.9 (9.5, 20.0)
LCS beverage	1.74 (1.59, 1.92)	1.58 (1.44, 1.74)	17.5 (13.6, 23.0)	1.73 (1.52, 1.97)	1.48 (1.28, 1.7)	28.9 (20.3, 40.4)	1.72 (1.55, 1.92)	1.6 (1.43, 1.78)	13.9 (9.2, 19.8)
LCS food	2.12 (1.76, 2.54)	2.09 (1.72, 2.54)	1.6 (−5.6, 8.8)	2.52 (1.80, 3.53)	2.39 (1.66, 3.46)	5.6 (−7.5, 22.8)	1.90 (1.54, 2.33)	1.88 (1.52, 2.32)	1.4 (−8.2, 11.3)
LCS tabletop	1.76 (1.58, 1.98)	1.61 (1.44, 1.82)	15.7 (10.1, 22)	1.90 (1.61, 2.23)	1.63 (1.37, 1.93)	24.0 (14.9, 37.0)	1.64 (1.40, 1.93)	1.54 (1.31, 1.81)	12.9 (5.7, 21.9)
									
*Number of LCS*									
1 vs none	1.63 (1.5, 1.76)	1.49 (1.37, 1.61)	18.5 (13.6, 24.3)	1.67 (1.49, 1.86)	1.47 (1.29, 1.66)	25.2 v16.6, 36.3)	1.56 (1.43, 1.7)	1.45 (1.33, 1.58)	16.7 (10.7, 25.3)
⩾2 vs none	2.66 (2.25, 3.15)	2.37 (2, 2.81)	11.6 (7.9, 16.2)	2.76 (2.16, 3.53)	2.17 (1.7, 2.76)	23.7 (15.8, 36.7)	2.54 (2, 3.23)	2.35 (1.85, 2.97)	8.7 (6.7, 11.4)
									
*Maintain (excluding individuals reporting intent to lose weight)*									
Total LCS	1.56 (1.43, 1.70)	1.48 (1.36, 1.61)	11.7 (7.9, 16.3)	1.56 (1.36, 1.78)	1.41 (1.23, 1.63)	21.7 (14.2, 34.7)	1.54 (1.38, 1.73)	1.49 (1.34, 1.66)	7.4 (2.3, 13.3)
LCS beverage	1.63 (1.46, 1.81)	1.53 (1.38, 1.71)	11.8 (7.1, 17.3)	1.53 (1.31, 1.78)	1.37 (1.16, 1.61)	25.8 (14.5, 44.7)	1.69 (1.47, 1.94)	1.63 (1.43, 1.87)	6.7 (2.0, 13.2)
LCS food	1.97 (1.51, 2.56)	1.96 (1.50, 2.55)	0.7 (−10.5, 14.1)	2.24 (1.44, 3.48)	2.05 (1.32, 3.17)	10.9 (−1.2, 31.9)	1.81 (1.31, 2.51)	1.82 (1.31, 2.51)	−0.6 (−6.2, 5.2)
LCS tabletop	1.69 (1.48, 1.93)	1.59 (1.39, 1.81)	11.7 (6.8, 18.6)	1.73 (1.38, 2.16)	1.56 (1.24, 1.95)	19.0 (9.2, 34.9)	1.65 (1.33, 2.04)	1.58 (1.29, 1.94)	8.3 (2.6, 16.8)
									
*Number of LCS*									
1 vs none	1.49 (1.34, 1.66)	1.42 (1.28, 1.57)	12.7 (7.8, 19.1)	1.52 (1.3, 1.77)	1.39 (1.18, 1.64)	20.3 (11.5, 38.9)	1.45 (1.27, 1.66)	1.4 (1.23, 1.6)	8.8 (2.8, 18)
⩾2 vs none	2.53 (2.09, 3.05)	2.37 (1.97, 2.85)	7.1 (3.3, 12.4)	2.27 (1.7, 3.04)	1.88 (1.41, 2.5)	23 (11.7, 40.9)	2.66 (2, 3.53)	2.57 (1.96, 3.38)	3.3 (−2.7, 10.4)

Abbreviations: BMI, body mass index; CI, confidence interval; LCS, low-calorie sweetener.

a Model 1: adjusted for age group, race/ethnicity and gender.

b Model 2: adjusted for factors from model 1 plus BMI in 6 categories (<18.5, 18.5–24.9, 25–29.9, 30–34.9, 35–39.9 and ⩾40 kg m^−2^).

c Proportion mediated estimated by estimating the percent difference of coefficients (log prevalence ratio) before and after adjusting for weight loss/maintenance intention. Bias-corrected 95% CI estimated using 500 bootstrap resamplings.
